# Khellin Mitigates Cisplatin-Induced Renal Injury by Targeting Oxidative Stress, Inflammation, and Apoptosis: Integration of Network Pharmacology, Molecular Docking, and Preclinical Validation

**DOI:** 10.3390/ph18060836

**Published:** 2025-06-03

**Authors:** Zeina W. Sharawi, Shimaa A. Abass, Manal A. Zubair, Rabab A. Hegazy, Foad A. Farrag, Abdelrahman Hamdi, Mohammed A. El-Magd, Abdullah A. Elgazar

**Affiliations:** 1 Department of Biological Sciences, Faculty of Science, King Abdulaziz University, P.O. Box 80203, Jeddah 21589, Saudi Arabia; zsharawi@kau.edu.sa; 2 Department of Biochemistry, Faculty of Pharmacy, Kafrelsheikh University, P.O. Box 33516, Kafrelsheikh 33516, Egypt; shimaa_abass@pharm.kfs.edu.eg; 3 Clinical Microbiology and Immunology Department, Faculty of Medicine, King Abdulaziz University, Jeddah 21589, Saudi Arabia; mzubair@kau.edu.sa; 4 Department of Biology, University College in Darb, Jazan University, P.O. Box 114, Al-Darb, Jazan 45142, Saudi Arabia; rhegazy@jazanu.edu.sa; 5 Department of Anatomy, Faculty of Veterinary Medicine, Kafrelsheikh University, P.O. Box 33516, Kafrelsheikh 33516, Egypt; foad.farrag@vet.kfs.edu.eg; 6 Department of Pharmaceutical Organic Chemistry, Faculty of Pharmacy, Mansoura University, Mansoura 35516, Egypt; abdelrahmanhamdi2012@yahoo.com; 7 Department of Pharmacognosy, Faculty of Pharmacy, Kafrelsheikh University, P.O. Box 33516, Kafrelsheikh 33516, Egypt

**Keywords:** acute kidney injury, nephrotoxicity, Khellin, cisplatin, oxidative stress

## Abstract

**Background/Objectives**: The present study aimed to evaluate the nephroprotective role of Khellin (Khe) against cisplatin (CDDP)-mediated nephrotoxicity in rats. **Methods**: We assessed oxidative stress markers (MDA, CAT, SOD, GPx, and iNOs), inflammatory markers (TNFα, IL6, IL10, and MCP1), apoptotic markers (Bax and Bcl2), and the renal damage marker (Kim1). Network pharmacology and molecular docking studies were performed. In vitro, Khe effects were tested on normal kidney cells (Vero) and liver cancer cells (HepG2) treated with CDDP. **Results**: Network pharmacology and docking suggested Khe’s activity primarily affects oxidative stress and inflammatory pathways, notably through MAPK14 and PI3K downregulation. In vitro, Khe reduced CDDP’s cytotoxicity in Vero cells while maintaining anti-proliferative effects on HepG2 cells. In vivo, CDDP significantly increased serum creatinine, urea, Kim1, oxidative stress markers (MDA and iNOS), and inflammatory markers (TNFα, IL6, and MCP1) while decreasing antioxidant markers (SOD, GPx, CAT, and SOD3) and anti-inflammatory cytokine (IL10) levels. Khe treatment dose-dependently attenuated these changes, with the 100 mg/kg dose showing the most significant renoprotection. Histopathological analysis confirmed improved renal tissue integrity in Khe-treated groups. **Conclusions**: This study demonstrates that Khe exerts significant nephroprotective effects against CDDP-induced nephrotoxicity by mitigating oxidative stress, inflammation, and apoptosis while improving renal function and structure. These findings suggest Khe as a promising therapeutic candidate for preventing CDDP-related kidney injury.

## 1. Introduction

Acute kidney injury (AKI) affects about 13.3 million and leads to 1.7 million mortalities annually around the world [1]. AKI was found to be associated with a high prevalence of chronic kidney disease and higher mortality [2]. Several conditions contribute to the occurrence of AKI, including drugs, eating habits, illnesses, ischemia, and infection [3]. Previous studies illustrate that drug-induced nephrotoxicity is the leading cause in about 60% of AKI cases in hospital settings [4]. Anticancer medications and chemotherapeutic agents have a significant role in drug-induced nephrotoxicity, and, therefore, their impacts on the treatment are probably restricted [5]. For example, although cisplatin (cis-diamminedichloroplatinum II, CDDP) was broadly used to treat several tumors [6], its usage has mainly been restricted due to its well-known complication, particularly AKI [7]. About one-third of CDDP-treated patients will develop AKI [8]. CDDP induces nephrotoxicity by activating apoptosis, oxidative stress, and inflammation [9,10,11]. Increased release of reactive oxygen species (ROS) emerges early after cellular uptake of CDDP, leading to increasing oxidative stress in renal tissues and promoting apoptosis and inflammation [12,13,14]. Magnesium supplementation and volume expansion have been used to avoid CDDP-mediated AKI [15], but their outcomes are still insufficient. Antioxidants and CDDP transport blockers can also alleviate CDDP-mediated AKI [16], but they have limited efficacy and are still far from satisfactory. Consequently, there is an urgent need to recognize new substances that could overcome CDDP-induced AKI.

Recent in vivo and in vitro studies illustrated that several natural products have specific anti-inflammatory, antioxidant, and anti-apoptotic activities that could control the pathways associated with CDDP-mediated renal damage [17]. Historically, *A. visnaga* was employed to alleviate symptoms associated with kidney stones and promote renal health due to its smooth muscle relaxant properties [18]. Beyond its traditional applications, *A. visnaga* has garnered scientific interest for its diverse biological activities, including antioxidant, anti-inflammatory, and vasodilatory effects [18,19]. These properties are attributed to the presence of Khellin (Khe) as a major furanocoumarin found in *A. visnaga*. Khe has been thoroughly studied due to its ability to mitigate oxidative stress and modulate inflammatory pathways associated with the pathogenesis of several diseases, such as cardiovascular, neurodegenerative, and metabolic diseases, and it has a potential role in preventing cellular injury [20]. Hence, Khe shows promise in protecting tissues from injury, particularly in conditions linked to oxidative and inflammatory stress [21].

Still, no previous studies illustrate Khe’s protective impact against CDDP-induced AKI. In the present study, we utilized robust network pharmacology tools and molecular docking to investigate Khe’s potential use in managing CDDP renotoxicity. In vitro and in vivo experiments were also carried out to validate the in silico findings and helped to transition the results to clinical practice.

## 2. Results

### 2.1. Network Pharmacology Analysis

**Figure 1:**
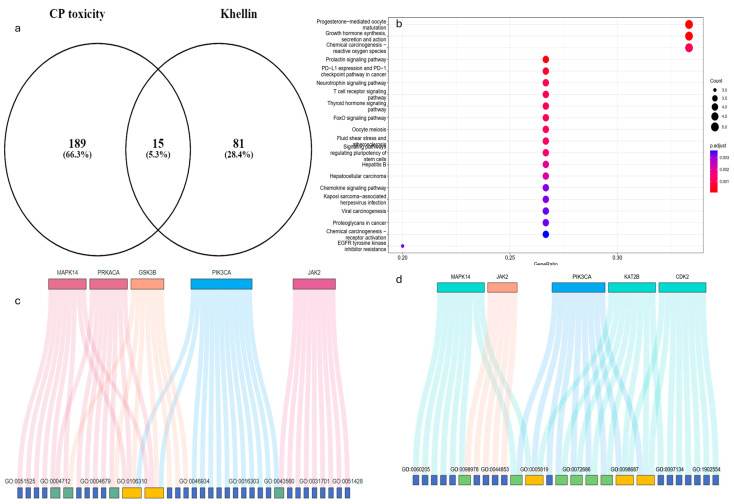
Network pharmacology analysis of the potential mechanism of Khe as a renoprotective agent and the relationship between Khe targets and CDDP-induced nephrotoxicity through multiple studies. (**a**) A Venn diagram highlights the overlap between CDDP toxicity-associated targets, and Khe shows 15 shared targets. (**b**) KEGG pathway enrichment analysis of the shared targets identifies significantly enriched pathways. (**c**) Gene Ontology (GO) analysis for molecular function (MF) links key genes. (**d**) GO analysis for cellular components (CCs).

The nephroprotective properties of Khe included fifteen protein targets discovered by network pharmacology (Figure 1a). KEGG pathway analysis (Figure 1b) highlights several enriched pathways associated with cellular injury mechanisms, two of the most notable being chemical carcinogenesis–ROS and prolactin signaling pathways. Integrating KEGG pathway analysis with gene distribution data identifies several critical genes, such as *PIK3CA*, *MAPK14*, and *GSK3B*, which are central to the PI3K/Akt and MAPK signaling cascades (Table 1). Enrichment analysis of biological processes (BPs) reveals the regulation of nucleocytoplasmic transport, peptidyl-serine phosphorylation, and the positive regulation of nucleocytoplasmic transport as major processes modulated by Khe (Supplementary Figure S1A). The molecular functions (MFs) enriched in this context, including protein serine/threonine kinase activity, insulin receptor substrate binding, and peptide hormone binding, strongly align with the KEGG findings (Figure 1c and Supplementary Figure S1B). Regarding cellular components (CCs), CDDP-induced injury significantly impacts the secretory granule, cytoplasmic, and vesicle lumen. These compartments are crucial for intracellular trafficking of signaling molecules and proteins, and their disruption affects cellular communication and homeostasis (Supplementary Figure S1C and Figure 1d). In summary, integrating KEGG pathways, BP, MF, and CC analyses provides insights into the potential of Khe nephroprotective mechanism and pinpoints PI3K/Akt and MAPK signaling pathways as the main targets.

### 2.2. Molecular Docking Analysis

**Figure 2:**
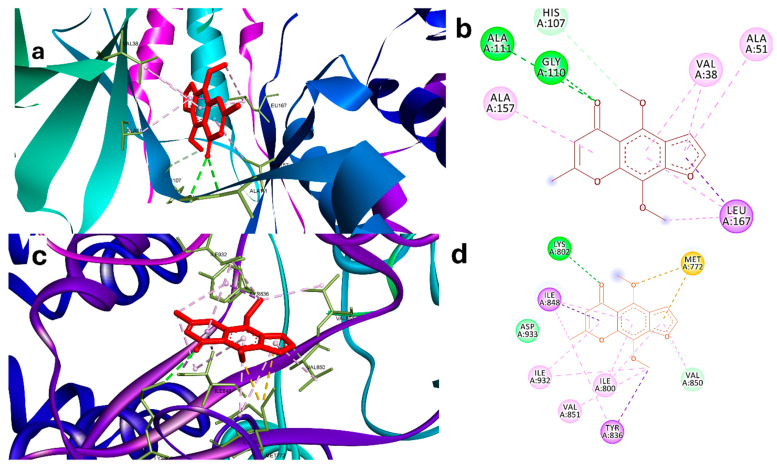
(**a**) Three-dimensional interaction of Khe in the binding site of MAPK14 (PDB:2qd9). (**b**) Two-dimensional interaction of Khe with residues of the active site of MAPK14. (**c**) Three-dimensional interaction of Khe in the binding site of PI3K (PDB: 3HHM). (**d**) Two-dimensional interaction of KHE with residues of active site of PI3K. Green dashed lines indicate hydrogen bonds, while purple dashed lines denote hydrophobic interactions. Blue dashed lines represent electrostatic interactions, and orange dashed lines correspond to sulfur interactions.

The validation of the software showed that RMSD is lower than 2, indicating good prediction ability of the software to recognize active poses (Supplementary Figure S2). Khe demonstrated a strong binding potential with MAPK14 through a network of interactions involving key amino acid residues (Figure 2a,b). Notably, the formation of conventional hydrogen bonds with HIS A:107 and GLY A:110 highlights the ability of Khe to establish stable interactions with critical residues within the active site, indicative of a strong binding affinity. Furthermore, the carbon–hydrogen bond with ALA A:111, and π-alkyl interactions with ALA A:157 and LEU A:167 contribute additional stability through hydrophobic interactions, essential for the proper orientation of the ligand within the binding site. Additional hydrophobic contacts with VAL A:38 and ALA A:51 further support the overall binding pose. The combination of these interactions likely plays a crucial role in the inhibitory activity of Khe against MAPK14, which is corroborated by its relatively favorable binding energy (ΔG = −25 kJ/mol) in comparison to the co-crystallized ligand (ΔG = −30.3 kJ/mol).

Khe also exhibited strong binding to PI3K, forming multiple interactions with key residues essential for the enzyme’s catalytic function (Figure 2c,d). Hydrogen bonds with LYS A:802 and MET A:772 were observed, underscoring the significance of these residues in stabilizing ATP’s phosphate group and ensuring proper coordination within the catalytic site. LYS A:802 directly engages with ATP during the catalytic process, while MET A:772 helps maintain the structural integrity of the binding region. Hydrophobic π-alkyl interactions with ILE A:848, ILE A:800, and TYR A:836 contribute to the stability and orientation of KHE in the active site. TYR A:836 assists in positioning substrates, while ILE A:848 and ILE A:800 form part of a hydrophobic pocket that influences substrate specificity. Additionally, interactions with VAL A:850, VAL A:851, ILE A:932, and ASP A:933 enhance the binding pose. ASP A:933 is particularly important, as it supports the coordination of magnesium ions required for ATP hydrolysis and reinforces the catalytic site structure. Together, these interactions disrupt PI3K enzymatic activity by interfering with its ability to phosphorylate substrates. This is further supported by the favorable binding energy of Khe (ΔG = −16 kJ/mol) in comparison to the co-crystallized ligand (ΔG = −18 kJ/mol), as presented in Table 2 and depicted in Figure 2c,d.

### 2.3. In Silico Prediction of Pharmacokinetic Properties of Khellin

Khe demonstrates several pharmacokinetic and safety properties that support its therapeutic potential, as shown in Table 3. It exhibits high intestinal absorption, with a human absorption probability of 0.999, indicating efficient uptake in the gastrointestinal tract and suitability for oral administration. This aligns well with its traditional oral use in treating conditions such as vitiligo and angina.

Its moderate oral bioavailability of 20 suggests that a clinically meaningful amount reaches systemic circulation, a favorable trait compared to many natural compounds with poor bioavailability. The compound’s plasma protein binding is moderate, at 82.68%, leaving about 17% unbound and therapeutically active, thereby reducing the likelihood of drug interactions due to displacement. Additionally, Khe shows a high probability (0.956) of penetrating the blood–brain barrier, which may be beneficial if central nervous system (CNS) effects are desired; however, further investigation is necessary.

Notably, Khe is not a substrate for the major hepatic enzyme CYP3A4, suggesting minimal risk of metabolic interactions associated with CYP3A4 variability. Its short half-life of less than three hours enables rapid systemic clearance, reducing the risk of accumulation and making it potentially suitable for acute dosing regimens. Furthermore, the compound carries a low risk of skin sensitization (probability: 0.487, low confidence), supporting its use in topical applications, particularly for vitiligo. Lastly, with an AMES mutagenesis probability of 0.316, Khe appears to have a low genotoxic risk, which is an important consideration for long-term therapeutic use.

### 2.4. Impact of Khe and/or CDDP on Vero and HepG2 Cells

**Figure 3:**
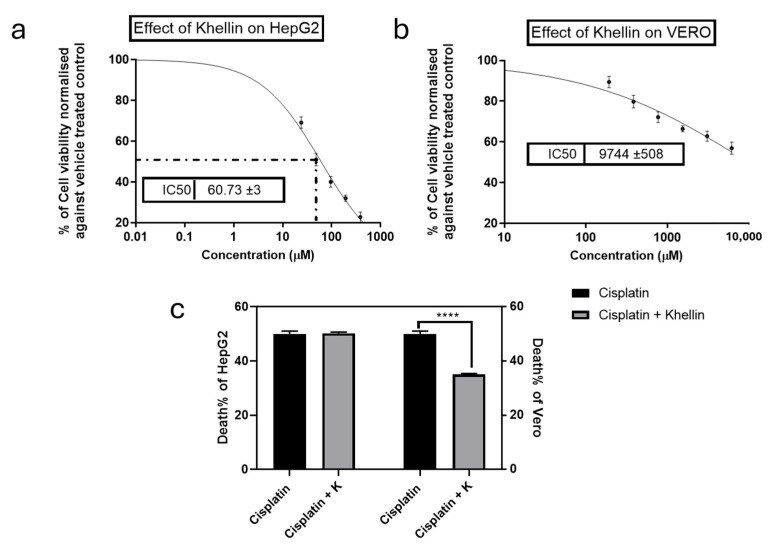
Effects of Khe on the proliferation of HepG2 (**a**) and Vero cells (**b**), as assessed by the MTT method. (**c**) The viability of HepG2 and Vero cells was compared to cells treated with CDDP alone, and the impact of combining Khe (38 µg/mL) with CDDP IC_50_ was also examined. The results are shown as the mean plus or minus the standard error of the mean (n = 3). **** *p* < 0.0001.

CDDP is well-known for its significant cytotoxicity on normal cells due to its low selectivity index. To evaluate whether Khe can mitigate this toxicity without compromising CDDP anticancer effects, an MTT assay was performed to determine the IC_50_ values of CDDP and/or Khe on liver cancer HepG2 and normal kidney Vero cells. CDDP exhibited IC_50_ values of 24.2 µM and 25.9 µM on HepG2 and Vero cells, respectively. In contrast, Khe displayed higher IC_50_ values of 60 µM and 9744 µg/mL on HepG2 and Vero cells, respectively (Figure 3a,b).

The protective effect of Khe against CDDP-induced cytotoxicity was assessed by co-treating the cells with CDDP at its IC_50_ and a subtoxic concentration of Khe (38 µM) to ensure Khe did not inhibit HepG2 or Vero proliferation. Khe reduced the cytotoxicity of CDDP on Vero cells by 30% compared to treatment with CDDP alone. Remarkably, the combination of CDDP with Khe did not alter the CDDP inhibitory effect on HepG2 cells, as evidenced by the unchanged proliferation rate (50.19%) compared to CDDP treatment alone (50%). These findings highlight Khe’s potential to significantly enhance the targeting of CDDP by protecting normal cells while retaining its anticancer efficacy (Figure 3c).

### 2.5. Effect of Khe Treatment on the Nephrotoxicity Markers

**Figure 4:**
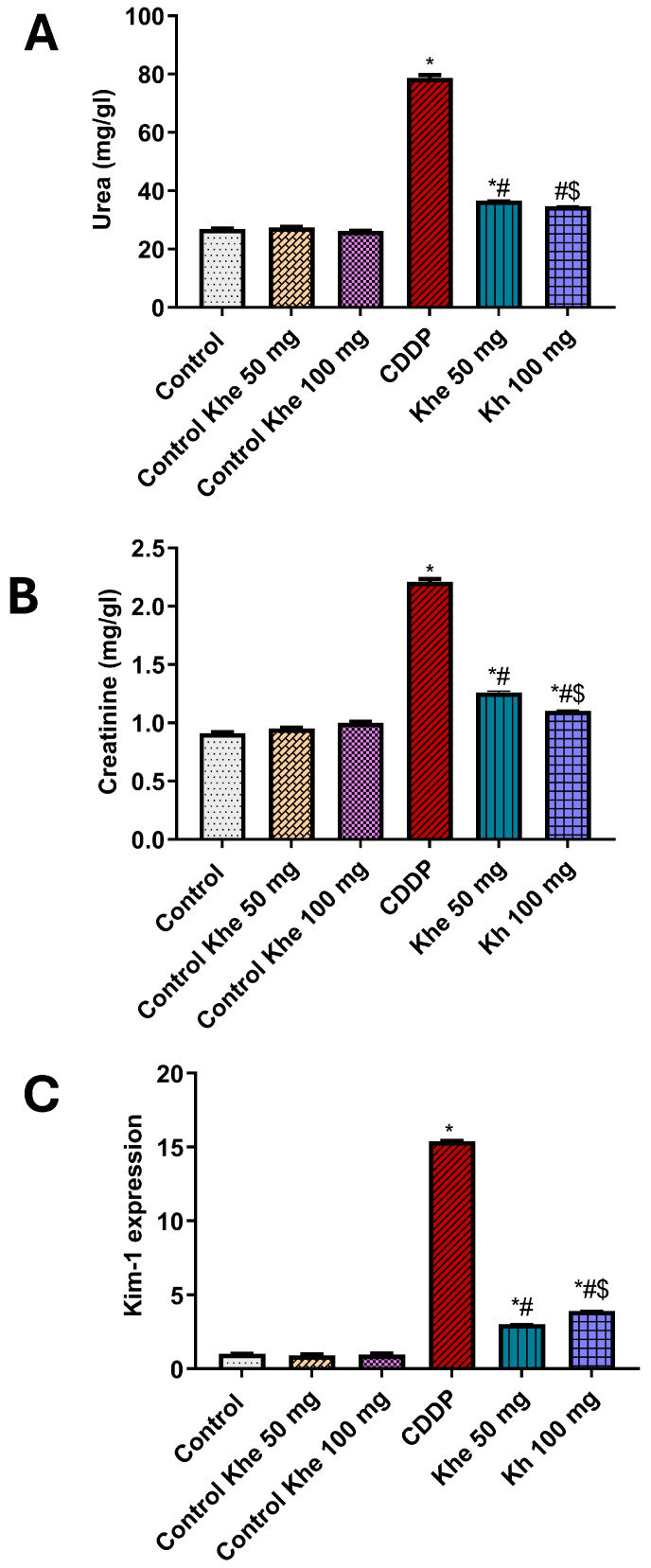
Impacts of CDDP and/or Khe on serum levels of (**A**) urea (**B**) creatinine and (**C**) Renal mRNA expression of *Kim-1* in different rat groups. Data were analyzed using one-way ANOVA, followed by the Tukey method, and expressed as mean ± SD (n = 6). *, #, and $ indicate significant differences between the control, CDDP, and Khe 50 mg groups, respectively. Khe: Khellin. CDDP: cisplatin.

The renoprotective impact of Khe treatment was assessed by measuring the serum levels of urea and creatinine and calculating the relative gene expression of the kidney-damage marker *Kim-1* (Figure 4). Rats injected with CDDP displayed a significant (*p* < 0.05) increase in serum urea and creatinine levels compared to the control rats. On the other hand, these levels were significantly (*p* < 0.05) decreased in the Khe-treated group in doses 50 or 100 mg/kg, with better results at higher doses, compared to the CDDP group. In parallel, the untreated CDDP group showed a significant (*p* < 0.05) increase in the renal gene expression of *Kim-1* compared to the control rats. The treatment with Khe showed a significant (*p* < 0.05) decrease in the CDDP-induced elevated *Kim-1* renal expression in a dose-dependent manner.

### 2.6. Effect of Khe Treatment on Renal Oxidative Stress and Antioxidant Markers

**Figure 5:**
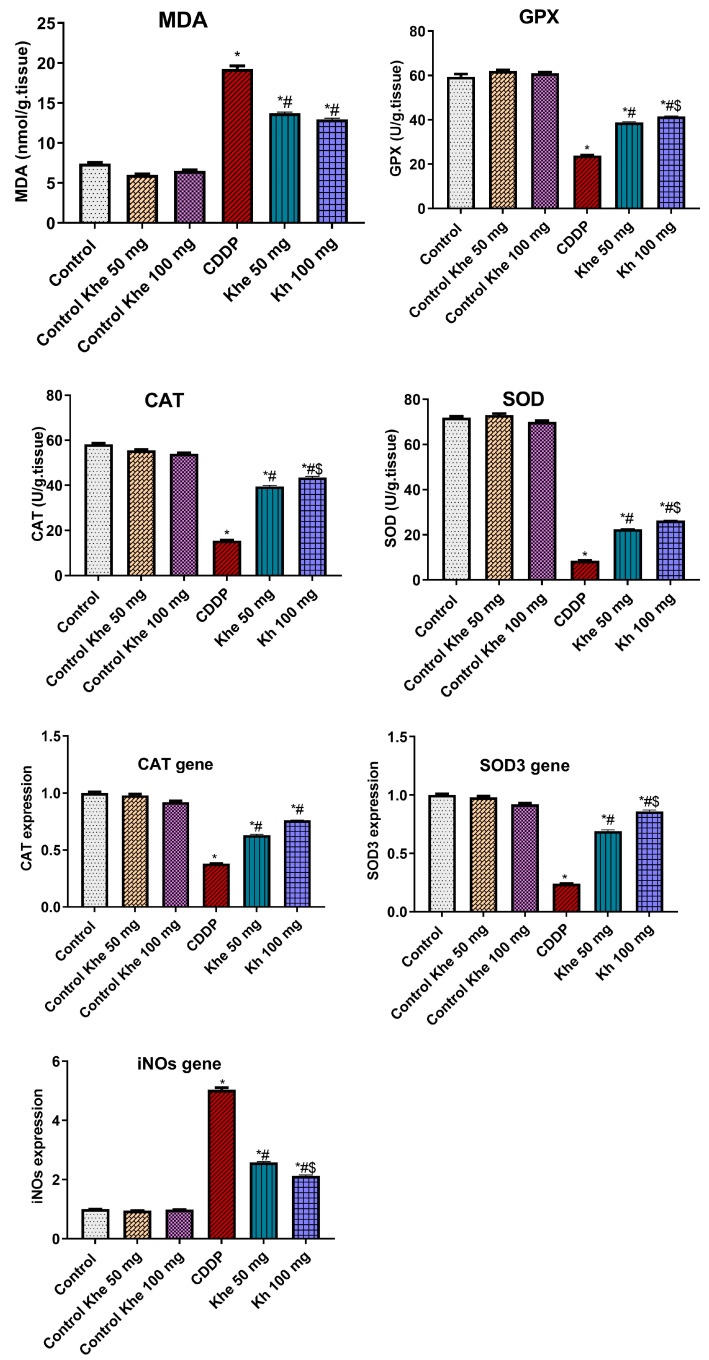
Effects of CDDP and/or Khe on the renal content of MDA; the renal activities of GPx, SOD, and CAT; and the renal gene expression of *CAT*, *SOD3*, and *iNOs* in different animal groups. Data are expressed as mean ± SD (n = 6). *, #, and $ indicate significant differences between the control, CDDP, and Khe 50 mg groups, respectively.

The CDDP-treated group exhibited a significant (*p* < 0.05) increase in the renal content of MDA compared to the three control groups (Figure 5). The renal content of MDA significantly (*p* < 0.05) decreased after Khe treatments (50 or 100 mg/kg) relative to the CDDP group. In contrast, the CDDP group showed a significant (*p* < 0.05) decrease in the antioxidant enzyme activities of SOD, GPx, and CAT compared to the control groups. On the other hand, Khe (50 and 100 mg/kg) administrations significantly (*p* < 0.05) increased the activities of these antioxidant enzymes when compared to the CDDP group. Moreover, the CDDP group showed a significant (*p* < 0.05) decline in the expression of *CAT* and *SOD3* and a substantial increase (*p* < 0.05) in *iNOs* when compared to the control groups (Figure 5). However, the treatment with Khe (50 or 100 mg/kg) significantly (*p* < 0.05) increased expression of *CAT* and *SOD3* and significantly (*p* < 0.05) decreased *iNOs* relative to the CDDP group. In all the previously mentioned parameters, treatment with 100 mg/kg Khe exhibited a more significant improvement than 50 mg/kg Khe (Figure 5).

### 2.7. Influence of Khe Treatment on Inflammatory Markers

**Figure 6:**
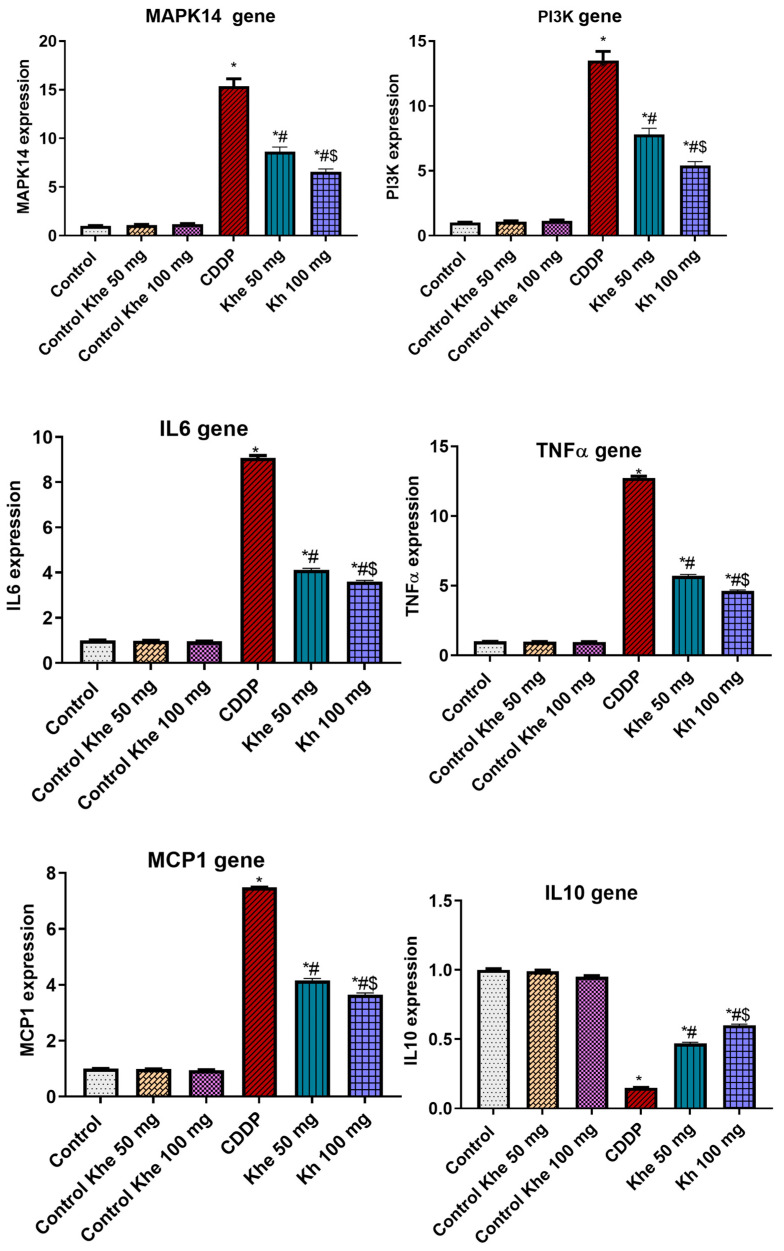
Impacts of CDDP and/or Khe on the expression of *MAPK14*, *PI3K*, *IL6*, *TNFα*, *MCP1*, and *IL10* genes in kidneys. Data are expressed as mean ± SD (n = 6). *, #, and $ indicate significant differences between the control, CDDP, and Khe 50 mg groups, respectively.

The effect of Khe treatment on the expression of inflammation-related genes (*MAPK14*, *PI3K*, *IL6*, *TNFα*, and *MCP1*) and the anti-inflammatory *IL10* gene was measured in the renal tissue (Figure 6). The CDDP group showed a significant (*p* < 0.05) upregulated expression in the five inflammatory genes and a significant (*p* < 0.05) downregulated expression in *IL10* compared to the control groups. On the other hand, the treatment with Khe (50 or 100 mg/kg) significantly (*p* < 0.05) reversed the increased levels of these inflammatory markers compared to the CDDP group. Among the two treated groups, Khe 100 mg/kg showed a better effect than Khe 50 mg/kg.

### 2.8. Impact of Khe Treatment on the Apoptotic Markers

**Figure 7:**
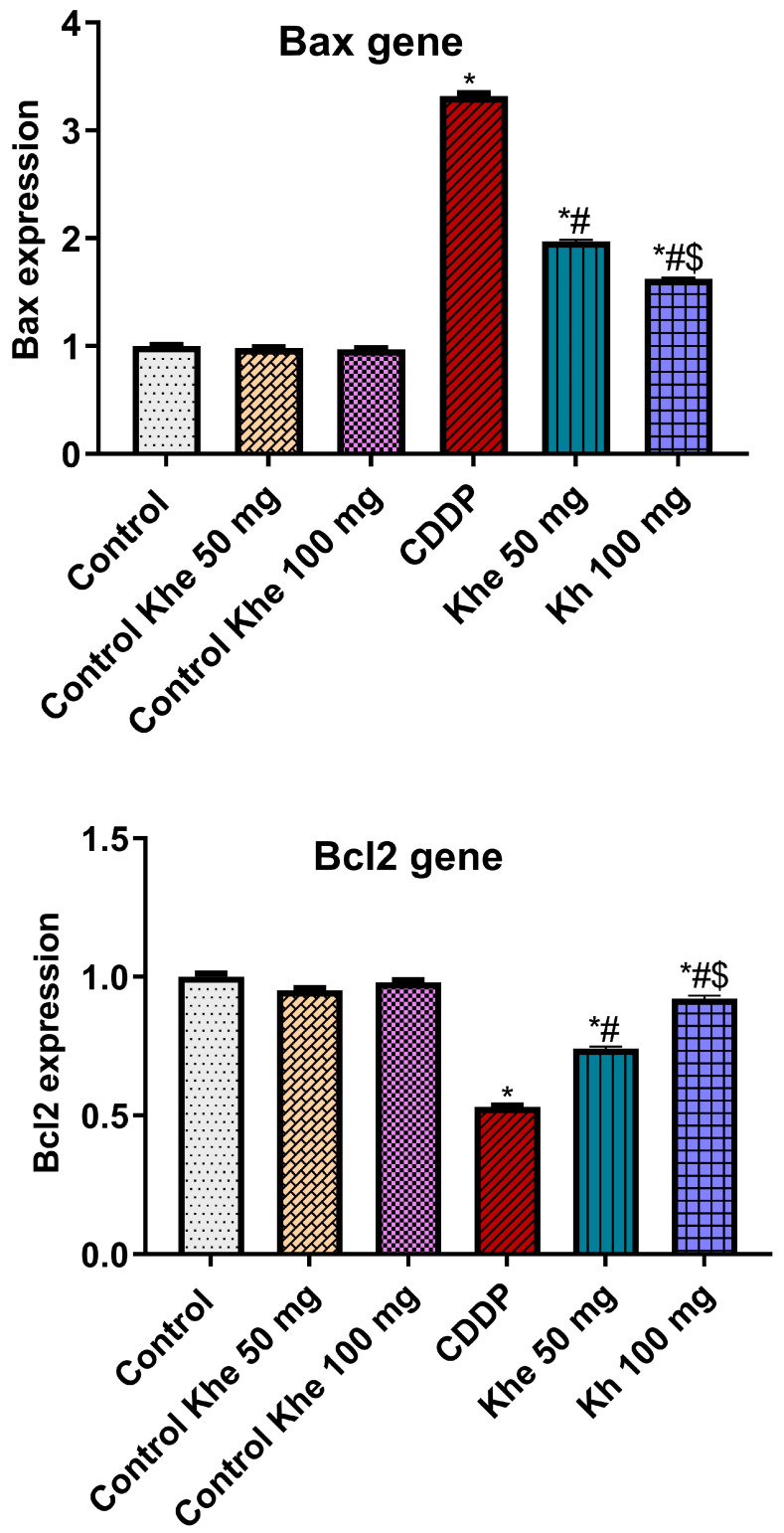
Effects of CDDP and/or Khe on the renal gene expression of *Bax* and *Bcl2* in different groups. Data are presented as mean ± SD (n = 6). *, #, and $ indicate significant differences between the control, CDDP, and Khe 50 mg groups, respectively.

The CDDP group exhibited a significant (*p* < 0.05) downexpression in the renal expression of the anti-apoptotic marker (*Bcl2*) and a marked overexpression in the apoptotic marker (*Bax*) compared to the control groups (Figure 7). On the other hand, the treatment with Khe (50 or 100 mg/kg) showed a significant (*p* < 0.05) decline in *Bax* and a significant (*p* < 0.05) elevation in *Bcl2*, with a better effect for 100 mg/kg Khe, compared to the CDDP.

### 2.9. Khe Decreased CDDP-Triggered Kidney Damage

**Figure 8:**
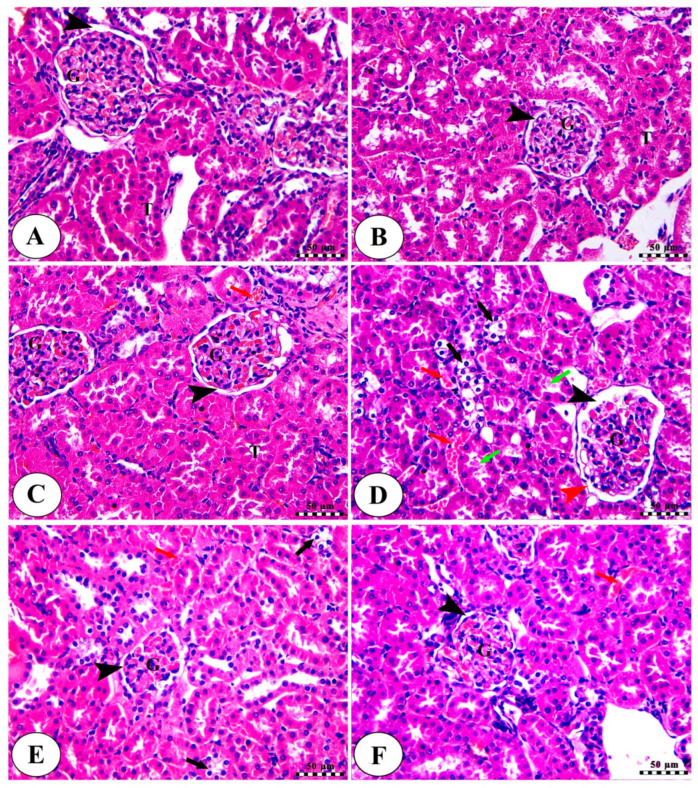
Kidney slices from the normal control group (**A**), the control Khe 50 group (**B**), the control Khe 100 group (**C**), the CDDP group (**D**), the Khe50 group (**E**), and the Khe100 group (**F**) were photographed under a microscope. H&E was used to stain all the slides; the scale bars are 50 µm. For more detailed information on the various labels featured in the images, please consult the main text.

The renal cortex of the three control groups (Figure 8A–C) showed intact renal corpuscle with intact renal glomeruli (G) and narrow capsular space (arrowhead), in addition to intact renal tubular epithelium (T). There was mild congestion of renal blood vessels (red arrow) in the control + Khe 100 group (Figure 8C). The CDDP group (Figure 8D) exhibited renal glomeruli with enlarged capsular space (black arrowhead), irregularities of Bowmann’s capsule (red arrowhead), vacuolar degeneration of renal tubules (black arrows), and accumulation of acidophilic proteinaceous substance inside the lumen of some tubules (green arrows), in addition to congestion of renal blood vessels (red arrows). The Khe 50 (Figure 8E) and Khe 100 (Figure 8F) groups displayed intact renal corpuscles with normal glomeruli surrounded by narrow capsular space (black arrowhead), mild congestion of renal blood vessels (red arrows), and mild vacuolar degeneration of some renal tubules (black arrows) in the Khe 50 group. The damage-score data indicated that the CDDP group sustained much more damage than the three control groups. However, the Khe-treated animals showed much less damage than the CDDP group (Table 4).

## 3. Discussion

The results obtained from KEGG pathways, BP, MF, CC, and molecular docking revealed that PI3K/Akt and MAPK signaling pathways are the primary targets of Khe to prevent CDDP-induced kidney damage. According to the KEGG pathway study, ROS is the primary mechanism by which CDDP induces AKI. The prolactin signaling pathway is essential in Khe’s nephroprotective role since dysregulation can increase cellular sensitivity to stress and damage [22]. KEGG pathway analysis and gene distribution data reveal critical PI3K/Akt and MAPK signaling pathway genes, including *MAPK14* (*p38 MAPK*) and *PI3K*. Gene expression and signaling systems essential for kidney cell homeostasis are altered by CDDP-induced nucleocytoplasmic transport disruption via MAPK14 activation [23,24]. CDDP dysregulation of bodily fluid levels highlights PI3K’s signaling role in fluid and electrolyte balance [25,26]. According to the BP enrichment study, Khe mainly affects peptidyl-serine phosphorylation, positive nucleocytoplasmic transport regulation, and control. Peptidyl-serine phosphorylation indicates MAPK-induced post-translational protein alterations that impact protein function and interactions. Kidney cells may compensate for CDDP effects by positively regulating nucleocytoplasmic transport via PI3K/Akt signaling [27]. On the other hand, the MF enrichment study showed insulin receptor substrate binding, peptide hormone binding, and protein serine/threonine kinase activity. MAPK cascades phosphorylate regulatory proteins involved in nucleocytoplasmic transport and gene expression via protein serine/threonine kinase activity. Hormone receptor binding enrichment significantly influences hormonal signaling, modulating kidney physiology and highlighting the PI3K/Akt and MAPK pathways [28]. CDDP-induced damage significantly damages the secretory granule, cytoplasmic vesicle, and vesicle lumens. They transport signaling chemicals and proteins intracellularly. Therefore, their disruption affects cellular communication and homeostasis. The ficolin-1-rich granule lumen and associated granules are also critical biological components because they may regulate MAPK and PI3K-mediated protein post-translational modifications [29]. Our molecular docking analysis also indicated that Khe bound well to MAPK14 and PI3K active sites. Those mentioned above in silico results were confirmed by qPCR findings, which showed that Khe, in a dose-dependent way, counteracted the higher *MAPK14* and *PI3K* expression caused by CDDP. Moreover, Khe is a furanocoumarin, a scaffold known to effectively bind ATP-binding sites of various kinases. Recent studies have explored Khe derivatives that specifically target p38 MAPK and PI3K, supporting our mechanistic hypotheses [30,31,32,33].

Regarding the pharmacokinetic properties of Khe, it is important to note that Khe has a well-established history of clinical use in treating various conditions, including skin disorders, asthma, angina pectoris, and hypercholesterolemia, at daily doses ranging from 20 to 200 mg [34,35], supporting its favorable pharmacokinetic and safety profile. Furthermore, our in silico pharmacokinetic analyses demonstrated properties consistent with previously reported data. These findings enhance the clinical relevance of our study.

The use of CDDP in clinics is usually associated with kidney damage and nephrotoxicity as the significant reported side effects [36]. CDDP and its metabolites, after their administration, are reabsorbed and secreted in the renal tubules and accumulate in the kidney [37]. The accumulation of CDDP and its metabolites causes acute kidney injury, possibly due to several pathways, including inflammation, cellular oxidative damage, vascular injury, proximal tubular cell damage, and apoptosis [38]. Accordingly, the CDDP-induced AKI causes alteration in the glomerular filtration rate of creatinine, uric acid, causing high levels of these renal function biomarkers in the blood during the clinical use of CDDP [39,40]. In this study, we confirmed that the intraperitoneal injection of CDDP caused nephrotoxicity as evidenced by marked elevation in serum urea and creatinine levels. In contrast, the therapy with Khe (50 or 100 mg/kg) showed a marked decrease in the serum urea and creatinine levels. A recent study also illustrated that pretreatment with *Ammi visnaga* seeds, which contain Khe as a major active constituent, could reverse the increase in kidney function parameters markers (creatinine, urea, and uric acid) in the Gentamicin-treated model of nephrotoxicity in rats [41].

Besides the kidney function parameters, histopathological changes in kidney tissues are also considered vital signs of kidney damage. CDDP administration causes deleterious structural variations in the kidney, including tubular vacuolization, accumulation of acidophilic proteinaceous substance inside the lumen of some tubules, and congestion of renal blood vessels. The administration of Khe at different doses protects against CDDP-induced deleterious changes in the renal tissue. Kim-1, a cell membrane glycoprotein, is a used biomarker for renal damage caused by ischemia or other nephrotoxic substances. Kim-1 acts as a receptor for apoptosis in cases of kidney tubular cell injury [42]. Due to the high expression of Kim-1 in injured renal tissues, it is considered a sensitive marker of nephrotoxicity [43]. Consistent with the formerly reported nephrotoxic murine models [40,44], we found that the administration of CDDP markedly increased the renal expression of *Kim-1*. The treatment with Khe markedly decreased renal *Kim-1* expression at both doses compared to the untreated CDDP group. Our study’s findings clearly illustrate Khe’s renoprotective effect against CDDP-induced kidney damage.

Cellular oxidative stress is an essential factor contributing to CDDP-mediated AKI’s pathogenesis [45]. The accumulation of CDDP in the renal tubular cells stimulates the generation of ROS, thus altering the oxidant–antioxidant system and harming renal tubular cellular functions and structures [46] by stimulating lipid peroxidation and depilating antioxidant enzymes [39]. Lipid peroxidation is a vital marker of oxidative stress in cells, causing damage to cell membrane lipids by generating ROS and increasing the level of MDA, which is considered the metabolic product of lipid peroxidation [47]. Additionally, an increase in iNOS and nitic oxide levels was also noticed during CDDP-induced kidney damage and toxicity [38]. Antioxidant enzymes, including CAT, GPx, and SOD, act as potent detoxifiers of peroxynitrite and lipid peroxides. These antioxidant enzymes play a role in various diseases associated with oxidative stress, including renal diseases [48]. Previous studies also reported the importance of ROS and their markers in CDDP-mediated kidney damage and its protection using natural antioxidants [12,36,40]. The current study also revealed significantly higher levels of renal MDA and *iNOs* overexpression and low renal activities of GPX, CAT, and SOD, and downregulated expression of *CAT* and *SOD3* genes in the CDDP group. However, the Khe treatment at both doses could significantly reverse these altered oxidative stress and antioxidant parameters. These findings suggest that Khe could protect the kidney oxidative damage mediated by CDDP by scavenging reactive oxygen species and maintaining catalytic antioxidant contents.

Besides cellular oxidative stress, activation of the inflammatory signaling pathway also has a vital role in the pathogenesis of CDDP-mediated nephrotoxicity [40]. IL6 plays a crucial role in the development of CDDP-mediated kidney damage [49]. The anti-inflammatory cytokine IL10 was downregulated upon CDDP administration [50] and protected the renal tissue against kidney ischemia and CDDP-mediated injury [51]. Several studies reported that TNFα is the key regulator of inflammatory responses, followed by CDDP administration, and is responsible for the upregulation of inflammatory chemokines and cytokines [40,52]. CDDP-treated animals showed upregulation of *TNFα* and *MCP1* in kidney tissues [40,53]. In the present study, we observed markedly high renal expression of *IL6*, *TNFα*, and *MCP1* and low renal expression of *IL10* in CDDP-treated rats, supposing the contribution of inflammatory cytokines in CDDP-mediated AKI by causing damage to renal tubular cells. Moreover, the altered renal expression of these inflammatory cytokines was reversed upon administering Khe at the two doses, thus suggesting its protective impact on renal damage by alleviating the CDDP-induced inflammatory activities.

ROS produced upon CDDP administration also activate *Bax*, which induces mitochondrial permeability transition, promoting cytochrome c’s release, and ultimately activates caspase-3 and promotes the apoptotic process [54]. Bcl2, one of the anti-apoptotic family members, contributes to the preservation of the mitochondrial function and structure, inhibiting the occurrence of mitochondrial permeability transition and preventing apoptosis [55]. CDDP activates *Bax* and downregulates *Bcl2* expression [40,56]. Similarly, we also found that the administration of CDDP upregulated the renal expression of *Bax* and downregulated the renal expression of *Bcl2*. However, the treatment with Khe could reverse the previous markers, suggesting the anti-apoptotic role of Khe in protecting against the renal damage induced by CDDP. Moreover, MAPK inhibition downregulated CDDP-induced inflammation and attenuated its renal anti-proliferative impact by preventing Bax activation and apoptosis [57]. Our model showed strong *MAPK14*, Bax downregulation, and *Bcl2* overexpression in rats co-treated with Khe and CDDP.

In summary, we selected two cell lines to establish a proof of concept that Khellin may exert a renoprotective effect against cisplatin-induced toxicity without compromising its anticancer efficacy. Vero cells were chosen for their widespread application in drug toxicity screening, while HepG2 cells were selected due to the common use of cisplatin in hepatocellular carcinoma treatment. To address the limitations of in vitro models—particularly their inability to replicate the complex physiological interactions present in whole organs—we complemented our experiments with comprehensive in vivo validation. While mechanistic insights were supported by network pharmacology and molecular docking, these approaches have inherent limitations. Molecular docking, for instance, can produce false-positive results and does not account for critical biological factors such as desolvation energy or pharmacokinetics. Therefore, further experimental validation is essential to confirm the predicted interactions between Khellin and its targets. Targeted experiments, such as gene knockout or overexpression studies, may be necessary to verify the involvement of the MAPK14 and PI3K pathways in human renal cell lines and in vivo models. This would facilitate the translation of our findings into clinical applications. Additionally, although Khellin has been used clinically to treat various conditions, its pharmacokinetic profile in cancer patients remains unclear. Further studies are warranted to assess its safety and determine whether dosage adjustments are needed for this specific population.

## 4. Materials and Methods

### 4.1. Ethics Statement

All procedures, including the use of animals and their care, were performed according to ARRIVE principles. All methods were carried out in accordance with relevant guidelines and regulations. The ethical approval for performing this experiment was obtained from the Kafrelsheikh University Animal Ethics Committee (KFS-IACUC/156/2023).

### 4.2. Network Pharmacology of Khe Targets

The 3D structure of Khe was obtained from the PubChem database (https://pubchem.ncbi.nlm.nih.gov/ (accessed on 15 February 2025)) in SDF format. Potential drug targets for Khe were identified using two bioinformatics tools: PharmMapper (http://www.lilab-ecust.cn/pharmmapper/ (accessed on 15 February 2025)), a pharmacophore-matching server, and the SwissTargetPrediction server (http://www.swisstargetprediction.ch/ (accessed on 15 February 2025)), which predicts targets based on chemical-structure similarity. PharmMapper and SwissTarget analysis identified 81 potential targets for Khe. The GeneCards database (https://www.genecards.org/ (accessed on15 February 2025)) was used to extract 189 molecular targets associated with nephrotoxicity. The common targets between Khe’s predicted targets and nephrotoxicity-related targets were identified using the Venny 2.0 online tool (https://bioinfogp.cnb.csic.es/tools/venny/ (accessed on15 February 2025)), generating a focused list of overlapping targets. Gene ontology (GO) enrichment and pathway analysis were conducted on the common targets to identify key biological processes (BPs), molecular functions (MFs), cellular components (CCs), and KEGG pathways linked to nephrotoxicity, as previously reported [58].

### 4.3. Molecular Docking of Khe Targets

To provide molecular insights, docking studies were performed with Khe and two of the most significant targets revealed by network pharmacology analysis, i.e., PI3K and MAPK14. The 3D crystal structures of PI3K (PDB ID: 3HHM) and MAPK14 (PDB ID: 2QD9) were retrieved from the Protein Data Bank (PDB, https://www.rcsb.org/ (accessed on 15 February 2025)). The protein structures were prepared for docking by removing water molecules, adding polar hydrogens, and optimizing protonation states using Discovery Studio Visualizer 2020. The Khe structure was prepared via energy minimization to optimize its conformation. Docking simulations were carried out using LeadIT 2.1 (BioSolveIT), employing the FlexX algorithm for accurate ligand–target binding predictions [59]. The software validation was confirmed through the redocking of the co-crystallized ligands to ensure the RMSD between experimental and docked pose was less than 2 Å. Multiple binding poses were generated and ranked for each protein–ligand complex based on binding energy and fit scores and inspected manually to select the pose achieving significant binding to amino acid residue involved in catalytic activity of the enzyme. The interactions within the ligand–protein complexes were visualized using Discovery Studio Visualizer 2020 to generate 3D and 2D interaction diagrams [60].

### 4.4. Pharmacokinetic Predication of Khe

The pharmacokinetic and toxicity profiles of the compound were predicted using the Deep-PK web platform (https://biosig.lab.uq.edu.au/deeppk/ (accessed on12 May 2025)). Deep-PK is a deep learning-based tool that utilizes graph neural networks and graph-based signatures to predict a wide range of ADMET properties.

The molecular structure of Khe was obtained in SMILES format from the PubChem database. This SMILES string was input into the Deep-PK server for analysis. The platform predicts ADMET-related properties, covering aspects such as absorption, distribution, metabolism, excretion, and toxicity. The results were compiled and analyzed to assess the compound’s pharmacokinetic and safety profile.

### 4.5. Preparation of Khe

*Ammi visnaga* L. fruits were obtained from a local market, Kafrelsheikh governorate, and identified by the Pharmacognosy Department, Faculty of Pharmacy, Kafrelsheikh University, and a voucher sample was deposited under number PG-1/1/2023. Khe was extracted from *A. visnaga*. In brief, dried and finely powdered fruits (1 kg) were first defatted using hexane in a Soxhlet apparatus, and the residue was subsequently extracted with 70% ethanol under reflux for 6 h. The ethanolic extract was concentrated under reduced pressure to yield an Semi-solid mass, which was triturated with hot water to selectively precipitate crude Khe. The precipitate was filtered, dried, and further purified using silica gel column chromatography with a hexane/ethyl acetate (7:3, *v*/*v*) elution system, affording 2 g of pure Khe as pale-yellow crystals. The isolated compound was characterized by its melting point using FALC melting-point apparatus (FALC Instruments, Trezzano sul Naviglio, Italy) and nuclear magnetic resonance (NMR) recorded on a JEOL ECA 500 NMR Spectrometer (JEOL Ltd., Tokyo, Japan) operating at 500 MHz ^1^H and 126 MHz ^13^C NMR, using CDCl_3_ as deuterated solvent and TMS as internal standard for chemical shifts. Chemical shifts (δ) were expressed in ppm with reference to the TMS resonance. It exhibited a melting point of 148–150 °C. ^1^H NMR showed characteristic signals at 7.64 (d, J = 2.3 Hz, 1H, aromatic proton), 7.03 (d, J = 2.2 Hz, 1H, aromatic proton), 6.17 (s, 1H, olefinic), 4.21 (s, 3H, OCH_3_), 4.07 (s, 3H, OCH_3_), and 2.42 (s, 3H, CH_3_) (Supplementary Figure S3). Meanwhile, ^13^C NMR showed peaks at 178.3, 164.2, 148.9, 147.4, 147.1, 145.6, 129.9, 119.4, 113.6, 110.6, 105.2, 62.4, 61.6, and 20.2 (Supplementary Figure S4). These results confirmed the identity and purity of the isolated compound as Khe, consistent with the literature-reported values [61,62].

### 4.6. MTT Assay

The MTT assay was used to evaluate the Khe cytoprotective effect against CDDP cytotoxicity. The normal kidney cell line (Vero) and the hepatocellular carcinoma cell line (HepG2) obtained from Vacsera (Egypt) were seeded at a density of 10^4^ cells per well in DMEM supplemented with 10% fetal bovine serum (FBS) and incubated under standard culture conditions. HepG2 cells were treated with CDDP and/or Khe for cytotoxicity evaluation at concentrations ranging from 0 to 200 µg/mL. Vero cells were treated with CDDP at concentrations ranging from 0 to 200 µg/mL, while Khe was tested at a broader range of 50 to 800 µg/mL. After 24 h of incubation, 10 µL of MTT solution (12 mM) was added to each well, followed by further incubation at 37 °C for 4 h. The resulting purple formazan crystals were solubilized in 100 µL of dimethyl sulfoxide (DMSO) per well and incubated for 30 min to ensure complete dissolution. The optical density (OD) was measured at 570 nm using a microplate reader (BioTek, Winooski, VT, USA), and the IC_50_ values for each compound in both cell lines were determined by fitting the data to a sigmoidal dose–response curve using GraphPad Prism 7 software. Once the IC_50_ values of CDDP and Khe were established, a protective assay was performed to evaluate Khe’s ability to protect normal Vero cells against CDDP-induced toxicity without diminishing CDDP’s cytotoxicity against HepG2 cells. Vero cells were pretreated with Khe at sub-cytotoxic concentrations for 2 h, and then co-treatment with CDDP at its IC_50_ concentration for 24 h was performed. Simultaneously, HepG2 cells were treated with CDDP at its IC_50_ concentration alone or in combination with Khe. Cell viability for both lines was assessed using the MTT assay, as described.

### 4.7. Animals and Treatments

Thirty-six Sprague Dawley male rats weighing 130 ± 10 g, obtained from the Egyptian Organization for Biological Products and Vaccines (Giza, Egypt), were allowed free access to a standard diet and water. They were kept at a temperature of 22 ± 2 °C, 12 h/12 h light/dark cycle, and humidity of 60%. After six days of acclimatization, all rats were randomly sorted into six groups. In the control group, rats received a single dose of saline (0.4 mL/kg) intraperitoneally (i.p.). In the control Khe 50 mg and Khe 100 mg groups, rats orally received 50 and 100 mg/kg Khe for three consecutive days [63]. In the CDDP group, rats were i.p. injected with a single dose of 7.5 mg/kg CDDP (50 mg/50 mL vials, EIMC United Pharmaceuticals, Cairo, Egypt) on the second day after acclimatization [40]. In the Khe 50 mg and 100 mg groups, rats orally received Khe 50 mg/kg or for Khe 100 mg/kg three consecutive days and were i.p. injected with a single dose of CDDP (7.5 mg/kg) on the 2nd day of treatment. At the end of the treatment period (2 days after CDDP injection), all animals were anesthetized using diethyl ether by inhalation. Blood samples were then collected from retro-orbital puncture of the eyes, left for coagulation for 30 min, and centrifuged at 4 °C at 1000× *g* for 15 min to separate serum. The serum gathered was kept frozen at −80 °C for further biochemical analyses. All animals were then euthanized by an overdose of anesthesia. The rat kidneys were excised, washed, weighed, and divided into three portions. The first part was fixed in buffered formalin (10%, pH = 7.4) for histopathology. The second part was rapidly dipped in liquid nitrogen and stored at −80 °C for real-time PCR. The last part was quickly homogenized in ice-cold phosphate-buffered saline to obtain tissue homogenate (10% *w*/*v*) for other biochemical measurements.

### 4.8. Biochemical Assays

The urea and creatinine concentrations in serum samples were measured calorimetrically according to Patton and Crouch’s [64] and Fossati et al.’s [65] methods, respectively, using commercial kits (Bio-Med, Cairo, Egypt). The homogenates of kidney samples from the different treatment groups were used for the assessment of MDA contents and activities of CAT, GPx, and SOD using an available assay kit (Biodiagnostic, Cairo, Egypt) and as previously described [66,67].

### 4.9. Real-Time PCR

The relative renal gene expression was determined in all rat groups using qPCR. TRIzol reagent (Invitrogen, Waltham, MS, USA, Cat# 15596026) was used for total RNA extraction from kidney tissues. A Nanodrop (Q5000, Quawell, San Jose, CA, USA) evaluated the isolated RNA purity and concentration. Reverse transcription was then performed using the available reverse-transcription kit. SYBR Green 2XMaster Mix (QuantiTect, Germantown, MD, USA) was mixed with the cDNA samples and primers (Table 5). We used the previously described thermal cycles [68]. The fold change in targeted genes was assessed using the Livak method (2^−∆∆Ct^). The internal control gene was *β-actin.*

### 4.10. Histopathology Examination

Kidney tissue samples isolated from rats were fixed in 10% buffered formalin at room temperature. Renal samples were then embedded in paraffin and sliced into 5 µm sections, which were then dewaxed in xylene and rehydrated using descending concentrations of ethanol. Staining using hematoxylin and eosin (H&E) was performed on kidney sections, and then the kidneys were examined using a light microscope.

### 4.11. Statistical Analysis

Data from the present experiment are presented as mean ± standard error of the mean (SD). Multiple comparisons were made using a one-way analysis of variance (ANOVA), followed by the Tukey–Kramer test for post hoc analysis. The criterion for significance was expressed using the 0.05 level of probability. All statistical analyses were performed using the Prism computer program (GraphPad Software Inc., version 8.0, San Diego, CA, USA).

## 5. Conclusions

The current study’s results illustrate Khe’s potential protective effect against CDDP-induced renal damage. This is possibly caused by modifying the kidney function-test parameters (urea and creatinine), renal oxidative damage, renal inflammatory activities, apoptosis, and restoring structural variations in the kidney. Thus, we suggest that Khe may be used as a potential supplement in the management of nephrotoxicity caused by CDDP treatment during cancer chemotherapy. Additional experiments are needed to support this study in clinical practice.

## Figures and Tables

**Table 1:** Gene distribution and correlation to KEGG enrichment and different biological processes (BPs), molecular functions (MFs), and cellular components (CCs).

Gene Symbol	Numbers Related KEGG	Numbers Related BP	Numbers Related MF	Numbers Related CC
PIK3	69/86	112/461	13/52	9/32
MAPK14	45/86	91/461	8/52	8/32
GSK3B	36/86	127/461	7/52	3/32
PRKACA	29/86	69/461	8/52	5/32
JAK2	19/86	174/461	9/52	5/32
CDK2	17/86	51/461	4/52	8/32
PTPN11	16/86	106/461	4/52	0/32
GSTT2B	6/86	0/461	0/52	0/32
NQO1	4/86	28/461	5/52	0/32
PLK1	4/86	78/461	4/52	4/32
KAT2B	4/86	65/461	7/52	8/32

**Table 2:** Molecular docking of Khellin and its binding energy in the targets.

Compound	MAPK14		PI3K	
	Binding Energy	RMSD	Binding Energy	RMSD
Standard inhibitor MAPK14	−30.3 kJ/mol	1.15	--	--
Standard inhibitor of PI3K	--	--	18 kJ/mol	1.5
Khellin	−25 kJ/mol	--	−16 kJ/mol	--

**Table 3:** Key pharmacokinetic properties of Khellin.

Category	Parameter	Prediction/Value	Interpretation
Absorption	Human intestinal absorption	0.999	Highly absorbed
	Human oral bioavailability (20%)	0.844	Bioavailable
	Human oral bioavailability (50%)	0.564	Bioavailable
Distribution	Blood–brain barrier Penetration	0.956	Penetrable
	Plasma protein binding (%)	82.68%	Therapeutic index < 90%
	Volume of distribution	0.59 L/kg	Low (primarily plasma-distributed)
Metabolism	CYP1A2 inhibition	0.991	Inhibitor
	CYP2C19 inhibition	0.883	Inhibitor
	CYP3A4 inhibition	0.839	Inhibitor
	CYP3A4 substrate	0.003	Non-substrate
Excretion	Half-life	0.255	Short (<3 h)
Toxicity	AMES mutagenesis	0.316	Non-mutagenic
	Skin sensitization	0.487	Low risk (low confidence)

**Table 4:** Scoring of histopathological lesions of renal parenchyma of different treated groups.

Groups	Control	Control Khe 50	Control Khe 100	CDDP	Khe 50	Khe 100
Glomerular affection	0	0	0	1.40 ± 0.10 ^a^	0.30 ± 0.01 ^b^	0.10 ± 0.01 ^c^
Degeneration of renal tubules	0	0	0	2.70 ± 0.14 ^a^	0.90 ± 0.04 ^b^	0.20 ± 0.01 ^c^
Congestion of blood vessels	0	0	0.30 ± 0.03 ^d^	2.50 ± 0.12 ^a^	1.00 ± 0.04 ^b^	0.50 ± 0.02 ^c^

(0) no lesion, (0.10–1.00) mild lesion, (1.10–2.00) moderate lesion, (2.10–3.00) severe lesion. Data are presented as mean ± SD (n = 6/group). Different superscript letters (a–d) in the row refer to significant differences at *p* ˂ 0.05. All groups were compared to each other.

**Table 5:** Primers used for the qPCR.

Gene	Forward Primer (5′-----3′)	Reverse Primer (5′-----3′)
*MAPK14*	GGAGATGAGCGTGAGAACGA	TCCAGGTCCTCATCTCCATC
*PI3K*	AACACAGAAGACCAATACTC	TTCGCCATCTACCACTAC
*KIM1*	TGGCACTGTGACATCCTCAGA	GCAACGGACATGCCAACATA
*iNOs*	CACCACCCTCCTTGTTCAAC	CAATCCACAACTCGCTCCAA
*SOD3*	AAGGAGCAAGGTCGCTTACA	ACACATCAATCCCCAGCAGT
*CAT*	GAATGGCTATGGCTCACACA	CAAGTTTTTGATGCCCTGGT
*TNFα*	GCATGATCCGCGACGTGGAA	AGATCCATGCCGTTGGCCAG
*MCP1*	TCGCTTCTGACACCATGCA	TGCTACAGGCAGCAAATGTGA
*IL6*	TCCTACCCCAACTTCCAATGCTC	TTGGATGGTCTTGGTCCTTAGCC
*IL10*	GTTGCCAAGCCTTGTCAGAAA	TTTCTGGGCCATGGTTCTCT
*Bax*	ACACCTGAGCTGACCTTG	AGCCCATGATGGTTCTGATC
*Bcl2*	ATCGCTCTGTGGATGACTGAGTAC	AGAGACAGCCAGGAGAAATCAAAC
*β-actin*	AAGTCCCTCACCCTCCCAAAAG	AAGCAATGCTGTCACCTTCCC

## Data Availability

The data supporting the present findings are in the manuscript.

## Supplementary Materials

The following supporting information can be downloaded at https://www.mdpi.com/article/10.3390/ph18060836/s1. Figure S1: Enrichment analysis results of renal injury-related targets in relation with Khe significantly enriched terms in, (A) Biological function (BP), (B) Molecular function (MF), and (C) Cellular component (CC); Figure S2. Validation of molecular docking by redocking the co-crystallized ligand A) Redocking of LGF standard inhibitor in the active site of MAPK14 (PDB:2QD9) where the experimental pose is yellow and the redocked pose is red. B) Redocking of wortmannin standard inhibitor in the active site of PI3K (PDB:3HHM) where the experimental pose is green and the redocked pose is red; Figure S3. ^1^H NMR spectrum of Khe; Figure S4. ^13^C NMR spectrum of Khe.

## Abbreviations

AKI	Acute kidney injury
Bax	Bcl-2-associated X protein
Bcl2	B-cell lymphoma 2
BP	Biological process
CAT	Catalase
CDDP	Cisplatin
CC	Cellular component
GPX	Glutathione peroxidase
IL6	Interleukin-6
iNOs	Inducible nitric oxide synthase
Khe	Khellin
Kim1	Kidney injury molecule 1
MAPK14	Mitogen-activated protein kinase 14
MCP 1	Monocyte chemoattractant protein 1
MDA	Malondialdehyde
MFs	Molecular functions
PI3K	Phosphoinositide 3-kinase
ROS	Reactive oxygen species
SOD	Superoxide dismutase
TNFα	Tumor necrosis alpha
