# Supplementary material for: Khellin Mitigates Cisplatin-Induced Renal Injury by Targeting Oxidative Stress, Inflammation, and Apoptosis: Integration of Network Pharmacology, Molecular Docking, and Preclinical Validation
Source: Pharmaceuticals (Basel). 2025 Jun 3;18(6):836. doi: 10.3390/ph18060836 (PMC12196209; doi:10.3390/ph18060836)
Supplement: Supplementary file 1 [file pharmaceuticals-18-00836-s001.zip › pharmaceuticals-3611401-supplementary.pdf]

Supporting information

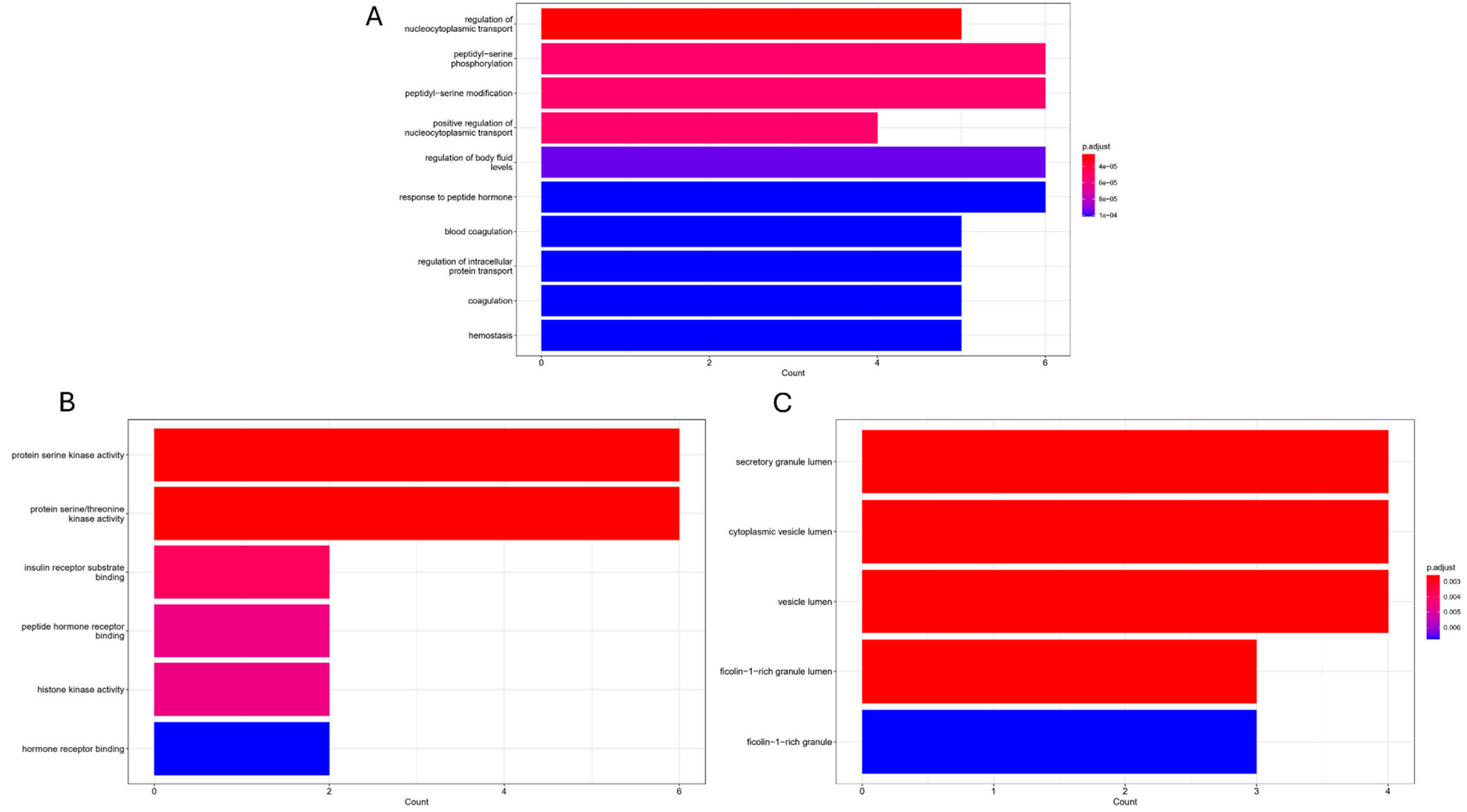

Figure S1. Enrichment analysis results of renal injury-related targets in relation with Khe significantly enriched terms in, **(A)** Biological function (BP), **(B)** Molecular function (MF), and **(C)** Cellular component (CC).

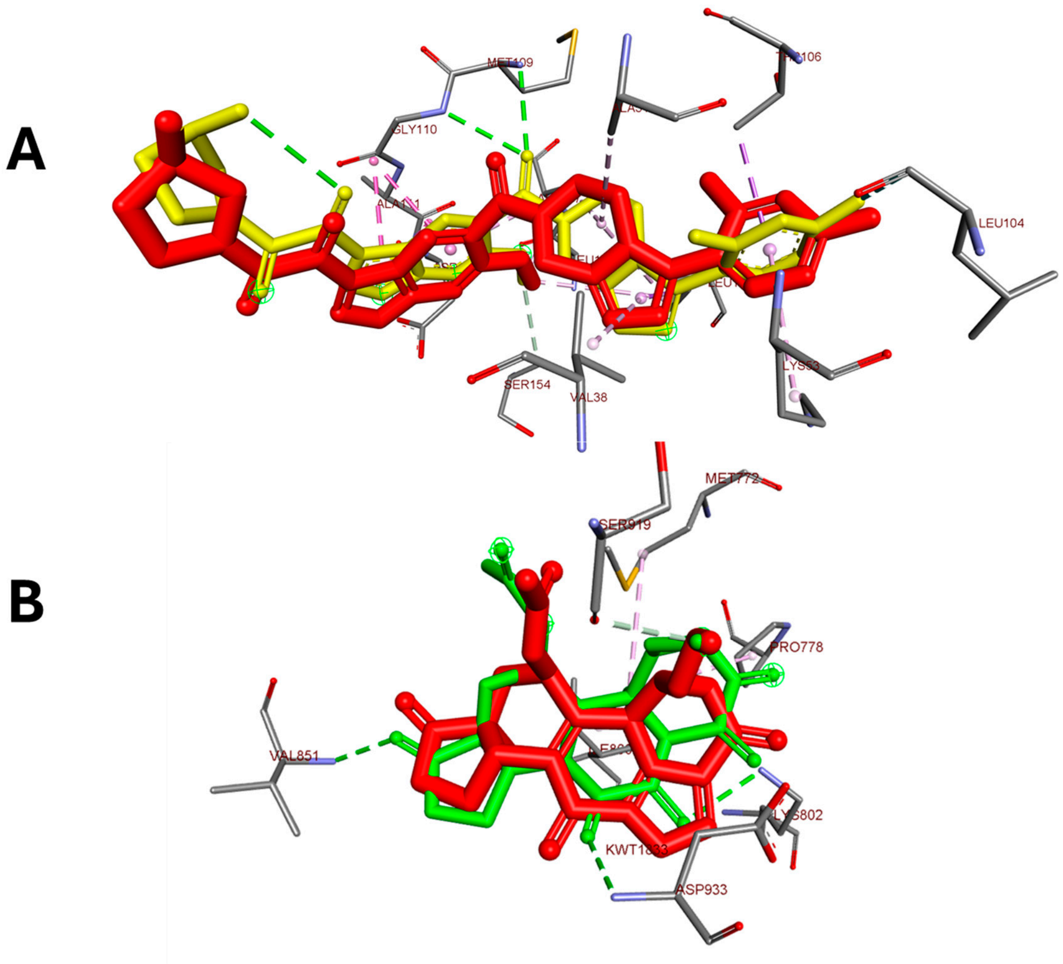

Figure S2. Validation of molecular docking by redocking the co-crystallized ligand A) Redocking of LGF standard inhibitor in the active site of MAPK14 (PDB:2QD9) where the experimental pose is yellow and the redocked pose is red. B) Redocking of wortmannin standard inhibitor in the active site of PI3K (PDB:3HHM) where the experimental pose is green and the redocked pose is red.

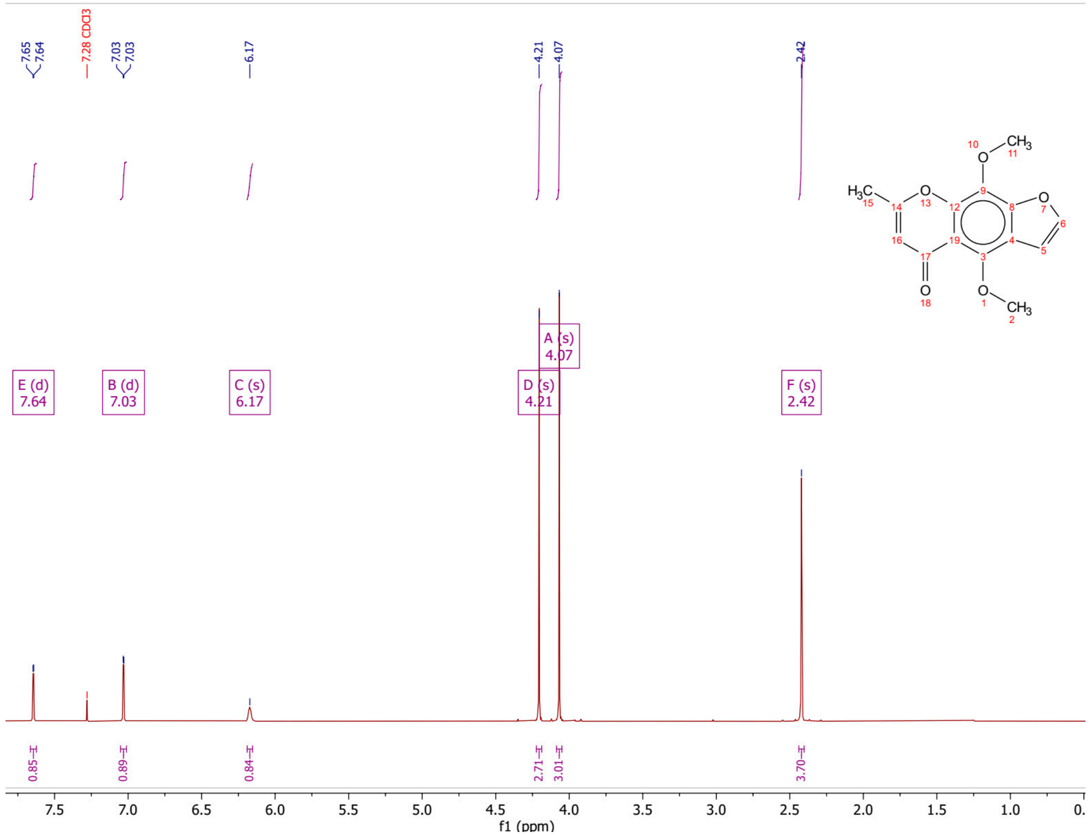

Figure S3. ^{1}H NMR spectrum of Khe.

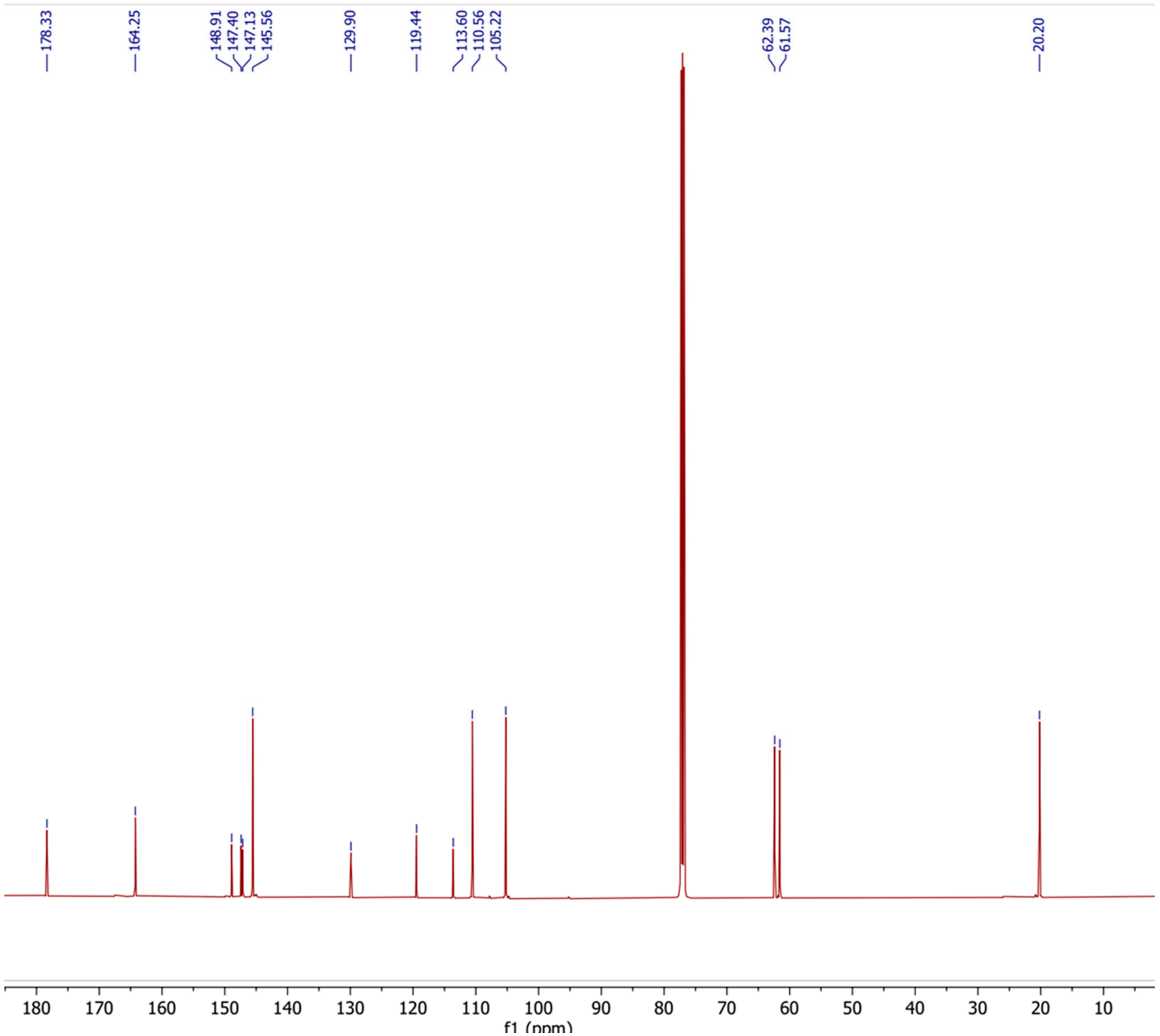

Figure S4. ^{13}C NMR spectrum of Khe
